# IgM nephropathy revisited

**DOI:** 10.5812/numonthly.2805

**Published:** 2012-09-24

**Authors:** Muhammed Mubarak, Javed I Kazi

**Affiliations:** 1 Histopathology Department, Sindh Institute of Urology and Transplantation, Karachi, Pakistan

**Keywords:** Kidney Diseases, Nephrotic Syndrome, Pathology

## Abstract

IgM nephropathy (IgMN) is an idiopathic immune complex-mediated glomerulopathy that was first described as a distinct disease in a nephropathology literature in 1978. Here, a historical review and the current status of IgMN in the light of world literature and the current experience will be presented. The Pubmed (www.pubmed.gov) search was made for articles on IgMN as the sole subject of the study or where it constituted a significant number of cases in a biopsy series in the world literature written in English. A total of 41 articles were found. A critical review of the literature was made. Soon after 1978, a series of reports were published mostly from the western world, but the interest in the entity did not withstand the test of time. No substantial basic medical research was carried out and the disease was largely ignored by the western researchers. More recently, a flurry of articles have appeared in the literature on the topic, mostly from tropical countries, and have renewed the interest in the entity. However, most of the current literature on IgMN is based on clinical observations, and experimental models and mechanistic studies of IgMN are lacking. There is an urgent need to develop consensus based criteria for the diagnosis of the condition, as well as, to focus the research on mechanistic studies to understand the pathogenesis of the disease better.

## 1. Introduction

IgM nephropathy (IgMN) is relatively a newly described, albeit controversial clinicopathologic entity which mainly presents an idiopathic nephrotic syndrome (INS) both in children and adults. This account reviews the definition of the disease, historical background, etiology, pathogenesis, pathology, clinical manifestations, treatment and the prognosis of the condition. It also highlights the need to develop a consensus based definition of the disease and calls for basic research to identify the causes and pathogenesis of the condition.

The Pubmed (www.pubmed.gov) search was made on IgMN articles as the sole subject of the study or where it constituted a significant number of cases in biopsy series in the world literature written in English. Following terms were used for the search: IgM nephropathy, renal biopsy, native kidney, and transplanted kidney. A total of 41 articles were found. The studies had been reported from all parts of the world, most of them from North America, Canada, Finland, Taiwan, Hong Kong, Middle East and South Asia. A critical review of the relevant studies was made which formed the foundation of this review along with researchers` experience with the disease.

## 2. Definition

The major controversy on IgMN has resulted from the lack of a universally acceptable definition of the entity. The disease, like IgA nephropathy (IgAN), is defined by its immunohistologic features: the presence of immunoglobulin M (IgM) as the sole or dominant immunoglobulin in the mesangial regions of the glomeruli in a diffuse (all glomeruli) and global (the entire glomerulus) distribution (1-4). However, there is no consensus on the minimum positivity of IgM required for the definition of the disease. Some authors have included renal biopsies showing only trace positivity of IgM in the IgMN category, others have included 1^+^, or 2^+^, positivity as the minimum threshold for the diagnosis of the disease (5-8). This has resulted in marked confusion and controversy in the literature on the unique nature of IgMN (1, 9). There is an urgent need to develop a consensus based definition of the condition. Since, both the light microscopy (LM) and electron microscopy (EM) findings are highly variable, the entire effort should be directed to develop standardization of the technique and the interpretation of immunofluorescence (IF) study for this purpose.

## 3. History

Although, the first formal reporting of IgMN in literature is widely credited to the two independent research groups led by Cohen (2), and Bhasin(3), who reported 12 and 11 patients, respectively, in 1978 presenting with heavy proteinuria, the predominant IgM deposits in the glomeruli, in fact, were first described in renal biopsies in 1974 by Putte et al.(10) in patients with persistent or recurrent hematuria (HU). Soon after 1978, a series of publications were reported from England, other parts of Europe, Canada, Japan, and Taiwan (4, 5, 8, 9). Interestingly, some of the largest and longest studies on the clinical course and natural history of the disease have been reported from Finland, Europe (11-13). More recently, when the interest in the disease has largely diminished in western countries, the disease is being reported more frequently from the centers in the developing countries (14-17).

## 4. Epidemiology

Like its definition, the epidemiology of the disease is fraught with controversies and confusion. There is no population based incidence or prevalence data on the disease in any parts of the world. Most studies on the incidence of the disease report the frequency of the disease in renal biopsies performed on a variety of indications, most commonly the idiopathic nephrotic syndrome (INS). The reported frequency of IgMN in literature has varied widely from 2% to 18.5% (1-3, 18). In fact, the frequency indicated a rising trend during the early period of its recognition (2-5). The first two pioneering studies reported incidence of 2% and 6.1% respectively in their biopsies (2, 3). These reports were soon followed by a study of 23 patients from England by Lawler et al. Who reported an incidence of 11.7% of IgMN (4). Hsu et al.(5) from Taiwan found a frequency of 10% of IgMN in all biopsies with primary glomerular disease. More recently, a frequency of 18.5% of IgMN in children with INS was found (1). Almost all the reported studies on IgMN relate to its occurrence in native kidneys; only occasional cases of its occurrence have been reported in renal transplant recipients (19).

The precise basis for the wide variations in the prevalence of IgMN is not known but it may be related partially to varying biopsy indications, varying case definitions used in different studies, and the genetic or environmental factors (5).

## 5. Etiology

As of today, no causes are known for the development of the primary form of IgMN. However, the deposits of IgM in the glomeruli may be seen in a variety of systemic diseases, such as systemic lupus erythematosus (SLE), rheumatoid arthritis, diabetes mellitus, paraproteinemia, and Alport’s syndrome (19, 20). The above conditions must be substituted by clinical and laboratory tests to diagnose primary IgMN.

## 6. Pathogenesis

An understanding of the disease mechanism is linked to etiology of the condition. For a disease like IgMN, where the etiology is unknown, the pathogenesis of the disease is also still largely unknown. Furthermore, very few studies have been conducted to elucidate the mechanism of this condition. The major reason for this apathy is the reluctance of the western researchers to accept this disease as a separate entity (9). Some studies have found elevated serum IgM or IgM immune complex concentrations in patients with IgMN (5, 21). However, no structural or biochemical abnormalities of IgM molecule, as observed in IgA immunoglobulin, have been described yet. The few studies on the co-localization of the complementary components along with IgM have suggested classical immune complex mediated activation of the complementary cascade, in the glomerular mesangium leading to injury and the reaction to the injury (5). This hypothesis is supported by the frequent presence of C1q and C4 deposits along with IgM in glomerular mesangium in the majority of cases, and the absence of properdin and factor B (5). Other studies, including the current one, have found C3 in the majority of cases , but C1q only infrequently (1). The source and the nature of the antigens triggering immune complex formation is not known, but it is hypothesized that certain antigens in the food or environment which preferentially induce IgM response may be the culprits. Abnormalities of T-lymphocyte regulatory role or a disturbance in the mesangial cell clearance of the passively trapped immune complexes have also been hypothesized (5). There are no animal models of the disease, which have hampered the study of the disease in the laboratory.

## 7. Pathology

The diagnosis of IgMN, like all other glomerulopathies, depends on detailed pathologic evaluation of renal biopsy by LM, IF, and EM. Of these, the first two are essential, and the EM is optional. It confirms the LM and IF findings. The pathologic findings need to be correlated with clinical and serological studies to exclude the secondary causes of IgM deposition. BOX shows the immunohistopathological features commonly observed on renal biopsies in patients with IgMN.

**Box:** Immunohistopathological Features and Their Prevalence in Cases of IgM Nephropathy on Renal Biopsies

**Light microscopic findings**
**Glomerular changes**
Minor changes, in variable number of cases
Mesangial proliferation of variable degree, in most cases
Focal segmental glomerulosclerosis, in variable number of cases
**Tubulo-interstitial compartment**
Tubular atrophy/interstial fibrosis of variabel degree, in variable number of cases
No tubular atrophy, in variable number of cases
**Vascular changes**
Mild fibrointimal thickening of arteries, in variable number of cases
**Immunoflourescence findings**
IgM, universal and defining feature of the disease
IgA (trace/minimal), variable, usually in minority of cases
IgG (trace/minimal), variable, usually in minority of cases
C3, variable, usually in upto half of cases
C1q, variable, usually in majority of cases in some studies
**Electron microscopic findings**
Minor changes or mesangialproliferation commensurate with light microscopic changes
Fusion of foot processes, proportional to degree of proteinuria
Electron dense deposits of variable density, a variable feature

### 7.1. Light Microscopy

**Figure 1:**
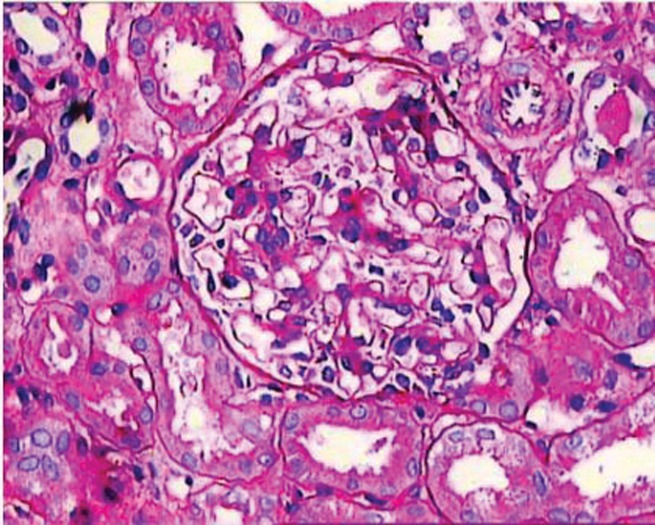
Medium-Power View Showing a Glomerulus With Mild Mesangial Hypercellularity on Light Microscopy Most of the mesangialregions exhibit upto four mesangial cell nuclei surrounded by mesangialmatrix. This is the most frequent morphologicmanifestation of IgMnephropathyon light microscopy. The surrounding tubules and a small arteriole are unremarkable. (PAS stain, ×200).

In contrast to the consistent deposits of IgM on IF microscopy, the LM findings in IgMN are quite heterogeneous, as in IgAN and lupus nephritis. The spectrum of morphologic alterations encompasses the whole spectrum ranging from minor changes, to variable degrees of mesangial proliferation, usually of mild to moderate degree, to focal segmental glomerulosclerosis (FSGS) pattern accompanied by adhesion formation with the Bowman’s capsule (1, 5, 13) (Figure 1). In a few reports small cellular crescents have also been observed (4, 22). A case of IgMN has been observed in a female 11-year-old child with full blown crescentic glomerulonephritis (CresGN) (unpublished data). The most prevalent morphologic alteration reported, consists of mesangial proliferation, mostly of mild to moderate degree (1, 5). In a minority of cases, severe mesangial proliferation with circumferential interpositioning of the expanded mesangium into the peripheral capillary walls with consequent splitting and tram-track appearance have also been reported (5, 22). The minor changes on LM are the next frequent pattern in most of the studies. It is difficult to distinguish this alteration from the minimal change disease (MCD) on LM examination alone and requires IF and EM to resolve the differential. This pattern has been reported in about one third of cases (1, 5, 13). FSGS pattern as the morphologic expression of IgMN is the most controversial feature. Many studies have excluded cases with this morphology from the IgMN category, while others have observed this lesion in a significant number of cases (1, 5). These cases indicate global mesangial positivity of IgM in contrast to nonspecific, segmental trapping of IgM in the idiopathic form of FSGS. The previous reports on prevalence of this morphologic pattern in biopsies of IgMN show wide variation (4, 5, 13, 18). The reported rates of this lesion have varied from 9 to 65.2% (1, 13). Focal global sclerosis is also quite common (4). Some researchers have noted progression of IgMN cases with minor changes or mesangial proliferation into FSGS on repeated biopsies in a variety of cases (13, 23).

Although most researches in progressive glomerular diseases are focused on glomerular alterations, studies have shown that alterations in the tubulointerstitial compartment are more important. Tubular atrophy and interstitial scarring are also commonly observed on renal biopsies of IgMN at the time of diagnosis and are usually mild (1, 13, 18, 20). Moderate and severe tubular atrophy have been rarely reported (1, 13, 20). Mild fibrointimal thickening of arteries has also been reported in a minority of cases (1, 13).

### 7.2. Immunoflourescence

**Figure 2:**
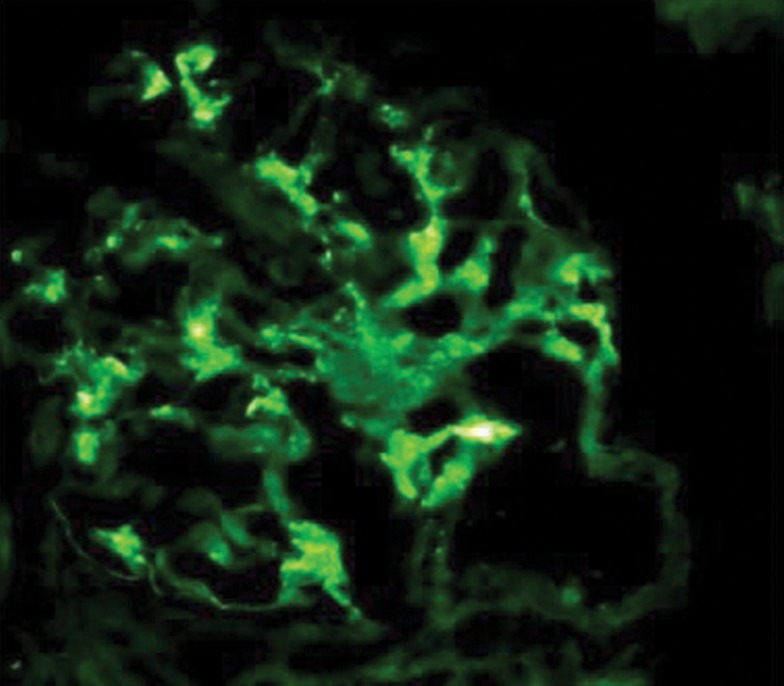
High-Power View Showing a Glomerulus With Diffuse Mesangial Positivity of IgM of an Intensity of 2+ on a Semi-quantitative Scale of 0 to 3+ on Immuno fluorescence Microscopy. (Fluoresced in isothiocyanate-conjugated IgM, ×400).

Like IgAN, IgMN is a diagnosis of IF microscopy. One of the characteristics of the disease is a diffuse and global mesangial positivity of IgM (either as sole immunoglobulin or predominant) of at least 1^+^intensityon a scale of 0-3^+^ (where 0 is absent, 1^+^ is mild, 2^+^ moderate, and 3^+^ is marked) (1, 13) (Figure 2). Some studies have included trace positivity of IgM on IF as IgMN (4). Concomitant but not dominant deposits of IgA and IgG are found in a small percentage of cases (1, 13). Complementary fragments of C3 and C1q are found in the majority of cases co-localized with IgM deposits (1, 13, 23).

### 7.3. Electron Microscopy

There are very few studies on the ultrastructural features of IgMN. In the majority of cases, no EM was done and the diagnosis was made solely on IF microscopy. The few studies that have carried out EM examination have noted small, granular to short linear, electron-dense deposits in the mesangium and paramesangium, along with variable degrees of mesangial cell proliferation and mesangial matrix expansion. Variable degrees of fusion of foot processes commensurate with the degree of proteinuria have also been observed (4, 5). The electron-dense deposits have typically been of low volume and low density, and in many cases, rather ill-defined (4, 5, 24).

## 8. Clinical Features

The clinical manifestations of IgMN are highly variable. The disease occurs predominantly in children and young adults but it can occur at any age. An early mean age of onset has been reported in males as compared with females in one study (18). Overall, the disease is slightly more common in males compared with females. However, some studies have found a female preponderance, especially in patients with HU (13).

Clinically, the disease most commonly presentswith idiopathic NS in both children and adults (1, 4, 5, 13). The disease frequently presents with HU or asymptomatic urinary abnormalities (AUA) (13). However, the majority of nephrologists around the world do not undertake renal biopsy in patients with the later manifestations, which accounts for the low frequency of patients with these manifestations. Isolated HU or proteinuria-hematuria (PU-HU) constituted almost half of biopsy indications in some previous reports (13). This may have implications for the severity of the pathological lesions observed on renal biopsy and the long term outcome of disease. Some authors have observed that patients with HU, especially adult females, have a good prognosis, compared with patients with NS or proteinuria (PU) (13). It has been observed, however, that IgMN in children are mostly presented as INS rather than HU or PU, as indicated by Myllimaki et al. (13), in which 32 of 36 children with IgMN presented as NS and only four children presented with non nephrotic proteinuria and none presented PU-HU or HU.

Hypertension is found in a significant minority of patients at presentation or biopsy (1, 4, 5). Its prevalence increases with increasing duration of the disease approaching 50% at 15 years of follow-up (13).

## 9. Natural History and Prognosis

The natural history and prognosis of IgMN, like its presentation and morphology, are also quite varied. There are multiple reasons for that. Apart from the variable length of follow-up of the patients, the lead-time diagnostic bias, and variable case ascertainment criteria are also partly responsible for this variable clinical evolution of the disease. The first two studies on IgMN did not report progression to ESRD, probably because of short follow-up period. The rate of ESRD development in the other reported studies has varied widely from four to 23 percent (13, 16, 20). The highest rate of 23 percent has been reported in the largest and longest follow-up study from Finland, in which renal failure was observed in 35% of cases in 15 years of follow-up, and ESRD developed in 23% of cases (13). These variable rates of ESRD development mostly reflect variable periods of follow up (20). The later authors also investigated for clinical and pathological prognostic factors leading to renal failure or ESRD. In multivariate analysis, hypertension was the only factor that predicted renal failure; on the other hand, none of the analyzed multiple factors were predicting ESRD. In the authors’ opinion this might have resulted from a small number of patients reaching ESRD in their study (13). An Indian study on adults and adolescents found that proteinuria and hypertension were predicting factors among the clinical and laboratory parameters, and FSGS, tubular atrophy and interstitial fibrosis among the pathological features (18). Another study identified microscopic HU, extent of mesangial proliferation, and global glomerulosclerosis as independent prognostic markers of multivariate analysis (25).

One important complication of IgMN is the transition of usual mesangial proliferative GN into the morphologic expression of FSGS. The later development can be only diagnosed in repeated biopsies and is associated with progressive disease. This complication has been studied only in a few reports (13, 23).

## 10. Treatment

Due to unknown etiology and pathogenesis of the condition, there is no specific treatment. There are also no randomized controlled trials on the treatment of IgMN. Corticosteroids constitute the mainstay of treatment as in MCD or primary FSGS (9). The steroid response pattern in IgMN varies considerably among the reported studies; the reported steroid resistance varies from 0 to 52% (9). The highest steroid resistance of 66% has been recently reported by Mokhtar (Saudi Arabia) (20). Border (9) reviewed nine affirmative papers on IgMN and found a mean steroid resistance of 28%. Steroid resistance was also found in about one third of the children with INS (1). The above studies indicate that IgMN responds less to steroids compared to typical MCD, and favors the hypothesis that IgMN is distinct from MCD (1, 13). Besides, not many investigators have found significant differences in clinicopathological or steroid response pattern among these diseases (24, 26-28).

There is little data on the use and response rates of immunosuppressive agents in patients with IgMN(9, 13). Oral cyclophosphamide has been used in a number of studies with response rates of up to 50% (13, 16). The data on cyclosporine usage is further scanty, with only occasional reports describing a favorable response in steroid dependent IgMN patients (16). Recurrent disease after renal transplantation has been successfully treated with anti-CD antibodies (rituximab), in combination with plasma exchanges, and immunoglobulins (29, 30).

## 11. Conclusions

In conclusion, IgMN is an important, but neglected cause of renal morbidity in both children and adults in many parts of the world. It shows a spectrum of morphologic changes ranging from minor changes to FSGS. Immunofluorescence is necessary for its diagnosis. Clinically, a poor response to steroid therapy distinguishes this disease from MCD. Further longitudinal studies are needed to clarify the status of the disease among the primary glomerulopathies. There is an urgent need for collaborative studies between health centers in the developing countries, where the disease is common, and the developed world, where resources and infrastructure for research are fully developed. Such studies can elucidate the etiology and pathogenesis of this disease and lead to development of effective therapeutic regimens.
